# Court-ordered forensic psychiatry treatment in prison: determinants of outcome and risk mitigation

**DOI:** 10.3389/fpsyt.2024.1436962

**Published:** 2024-09-03

**Authors:** Kerstin Weber, Sandrine Morier, Christophe Menu, Philippe Bertschy, François R. Herrmann, Panteleimon Giannakopoulos

**Affiliations:** ^1^ Division of Institutional Measures, Medical Direction, Geneva University Hospitals, Geneva, Switzerland; ^2^ Department of Psychiatry, University of Geneva, Geneva, Switzerland; ^3^ Secured Penitentiary Curabilis, Department of Institutions and Information Technology, Puplinge, Switzerland; ^4^ Department of Institutions and Information Technology, Geneva, Switzerland; ^5^ Department of Rehabilitation and Geriatrics, Geneva University Hospitals and University of Geneva, Thônex, Switzerland

**Keywords:** court-ordered treatment, forensic therapeutic community, mentally disordered offenders, prison, violence risk

## Abstract

**Background:**

Court-ordered forensic psychiatry treatments (COT) are specifically designed to reduce the risk of violence in mentally disordered offenders. Given their high costs and ethical issues, mental health professionals need admission criteria to be able to select those candidates with optimal benefit. This study analyses offender-related and treatment-related determinants of COT outcome and risk mitigation.

**Methods:**

This two-year longitudinal study assessed the evolution of 117 adult offenders admitted to a specialized medium-security forensic psychiatry clinic. Treatment outcome included court-ordered discharge locations and the Historical Clinical Risk Management (HCR) score evolution. Treatment progress was assessed every six months across five time-points including measures of protective factors, work rehabilitation and security. Outcome determinants included psychiatric diagnosis and type of offence.

**Results:**

Discharge locations are predicted by pre-treatment risk level. Lower HCR scores are associated with discharge into low-security psychiatry wards independently of the psychiatric diagnosis. Risk reduction follows diagnosis-specific and offense-related patterns and reveals that mentally disordered offenders with Cluster B personality disorders or those sentenced for drug crimes are significantly less prone to benefit from COT.

**Conclusions:**

Our findings indicate that criminological characteristics at baseline as well as diagnosis of personality disorders are the main determinants of treatment outcome in our care setting. Inmates with concomitant higher violence risk at baseline and presence of Cluster B personality disorders might benefit the least from court-ordered forensic inpatient psychiatric care in prison.

## Highlights

- Offenders with psychotic disorders benefit most from court-ordered forensic psychiatric inpatient treatment.
- Pre-treatment violence risk is the core predictor of forensic psychiatry treatment outcome independently of the nature of offenders’ diagnosis or crime.
- Patterns of risk mitigation depend on psychiatric diagnosis and type of offense in mentally disordered offenders.

## Introduction

Forensic mental health services are designed to provide treatment for individuals with severe and often disabling mental disorders and offending behaviors (1). Psychiatric care of mentally disordered offenders (MDO) produces better outcomes than incarceration in prison alone, yet forensic-psychiatric institutions are high-cost, low volume services which pose significant ethical problems since the length of stays are often long and even indefinite (2). Seclusion is a strong restriction of the individual’s freedom, and in long-stay patients, the shift becomes less on treatment and more on care and quality of life (3). Indeed, the purpose of forensic psychiatric care in detention is twofold: care and treatment for the patients and their individual well-being on one hand, and protection of the society from harm from the offenders on the other hand. This dual role is not only continuous dilemma for professionals, but also rises a societal challenge (2). The intensity, nature, and setting of these treatments are highly variable according to each country’s legal framework, mental health system, and political-economical support (4, 5). In Europe, in countries like the United Kingdom or Ireland, forensic psychiatric treatments are ruled by criminal law and the justice system, while in France, court-ordered psychiatric treatments are ruled by civil law and the health system. Most European countries provide forensic psychiatric treatments in specialized psychiatric clinics, yet some countries like Belgium, Greece, and Switzerland, also offer these treatments in prison (2, 5, 6).

In Switzerland, the Swiss Criminal Code makes a clear distinction between penalties and court-ordered treatments (COT), referred to as therapeutic measures. The latter are ordered when a penalty alone is not sufficient to control the risk of recidivism and the offender requires treatment in the interest of public safety. Therapeutic measures can be pronounced in conjunction with a custodial sentence, or against offenders who are criminally irresponsible and cannot be sentenced to a penalty. The decision of the Court is based on a psychiatric expert assessment to provide an opinion on the prospect of success of the treatment, the nature of the disorder-offense association, the probability of future offences, and the implementation of the measure. Measures include inpatient COT of mental disorders or addictions, outpatient treatments, or lifelong imprisonment. They are reviewed regularly according to the best interest of both the individual and public safety, because their duration can by far exceed the corresponding penalty which typically determines the duration of imprisonment (7). Recommendations for best practice of forensic-psychiatric care have suggested that the treatment milieu should include the therapeutic use of security, multidisciplinary working, as well as patient empowerment. Interventions designed to enhance motivation and engagement are of particular importance for MDOs (2). Forensic therapeutic communities have shown to reduce risk of recidivism through the development of a sense of belongingness and the capacity of responsible agency (6).

Given the complex and costly nature of forensic psychiatric treatment, it is essential to assess their outcome and efficiency of their impact on offending and antisocial behavior. The specificity of forensic psychiatric treatment is the need for risk awareness, which plays a key role in clinical and court decision making (1). Criteria for admission and discharge in security forensic psychiatry clinics are of legal nature. Rather than focusing on mental disorder recovery, forensic treatment outcomes include mitigation of risk (8, 9). Plus, previous evidence stressed the need to combine risk assessment with the investigation of protective factors, when planning discharge from secure forensic psychiatric units (10). The likelihood of recidivism is a core factor that determines the treatment success and is usually based on the structured assessment of dynamic risk and protective factors using *ad hoc* scales (11).

To limit prison seclusion and maximize violence risk management, forensic psychiatry professionals need to identify those MDO who have the best chances to use forensic care to be released from prison and continue psychiatric treatment in outpatient settings. In Switzerland, since 2006, a first institution for therapeutic measures hosts young adults aged 16 to 25 years in canton Zurich, offering a court-ordered inpatient social-therapeutic program with offense-oriented treatment and vocational training (12). Since 2014, a second specialized medium-security forensic psychiatry clinic (referred to as “Curabilis”) is located within the central prison of Geneva city and offers intensive inpatient court-ordered psychiatric treatment to adult mentally disordered offenders. As previously detailed, its hybrid carceral-medical management facilitates the coordination between mental health and prison professionals and allows for disease management, criminal desistance, and psychosocial rehabilitation in MDO (7). The discharge location at the end of the hospital stay, is determined by the Swiss court according to the MDO’s reduction of risk of violence.

Our previous cross-sectional study revealed that the median length of stay in Curabilis was of 2.5 years and that discharge depended on age, and presence of personality disorders or sexual offenses (7). The present observational cohort study is based on a 2-year follow-up of treatment progress parameters obtained from medical and criminological files and analyses their impact on discharge location and violence risk change. Treatment progress parameters include protective factors, criminological characteristics, as well as psychosocial rehabilitation (work attendance, social support network, escorted leaves) and therapeutic security (disciplinary measures, drug abstinence tests) variables. We hypothesize that discharge locations and violence risk decrease depend on a combination of type of psychiatric diagnosis and nature of offense.

## Materials and methods

### Treatment setting

Treatment interventions are inspired by the *forensic therapeutic community* approach, and the goal is desistance from crime and reduction of offending. Each of the 5 wards hosts 15 to 16 inpatients in a discrete wing, with its own collection of cells, group, dining, and living and therapy rooms, as well as staff offices. Daily program is organized based on community meetings attended by all inmates and mental health and prison professionals, small therapy, and creative and recreational activity groups, individual psychiatric-psychotherapy sessions, and psychotropic medication, as well as prison activities such as exercise, work, and education. In each ward, a multidisciplinary team of forensic mental health professionals (psychiatrists, psychologists, mental health nurses, movement, and occupational therapists) closely works together with prison officers, legal and social workers, chaplains, as well as education teachers and vocational trainers (leading laundry, cleaning, bakery, cooking, and gardening workshops). The forensic therapeutic community philosophy is grounded in an explicitly relational paradigm, using social skills training and interpersonal approaches, to allow for the acquisition of prosocial models of thinking (7, 13–15). Therapeutic communities offer a unique social climate, in which enabling social relationships and therapy sessions promote psychological health, by constructing a self-narrative that allows MDOs to define themselves in ways other than having a primary identity as an offender (14).

### Study design

This is a prospective naturalistic observational cohort study assessing treatment as usual over two years, using a range of data routinely collected in forensic psychiatry and prison practice. We extracted the data of all inmates consecutively admitted in Curabilis between August 2019 and August 2021, for court-ordered treatment (COT) according to Swiss Criminal Code (art. 59 SCC). After deduction of the 8 inmates who had refused to give informed consent, the final sample included 117 MDO. Repeated measures of each variable were extracted every six months across five time-points.

Data extraction was performed by two senior legal psychologists, trained in swiss forensic law procedures and not involved in the care programs of MDO cases, who conducted case file reviews to extract demographic characteristics (age, civil and educational status, nationality), PCL-R ratings, and psychiatric diagnosis as documented by expert assessments, which are part of each legal investigation in MDO according to the Swiss criminal procedure. These diagnoses were further confirmed by two independent fully trained psychiatrists according to ICD-10 criteria at admission in Curabilis. Cases with multiple diagnoses or offenses were considered in each diagnostic/offense category separately. Likewise, both psychologists extracted criminal data (offenses, COT duration, discharge location) from the court sentences, and total scores of violence risk HCR-20 and protective factors SAPROF scales. As part of the clinical progress monitoring, these scales are routinely assessed every six months by the senior forensic psychiatrist in each ward. The detailed information on previous psychiatric history, substance misuse, legal status, and criminal history available for each inmate allow for performing a meaningful observer rating. Finally, the study psychologists extracted the participation rates in rehabilitation programs and therapeutic security actions from each participant’s prison file as detailed below.

### Outcome measures

The main treatment outcome is the discharge location. All discharge locations are decided by the Court based on an independent assessment of dangerousness and risk of recidivism made by a psycho-criminologist, who is not involved in the care programs (7). To formulate is/her expert recommendation, the psycho-criminologist takes into account the therapist’s report on the MDO’s progress. He/she personally visits the MDO in Curabilis to perform a comprehensive offense-oriented interview as well as independent structured professional judgment ratings, and delivers a low/medium/high risk appraisal to the legal authority in charge of the COT supervision. In case of minor risk, the court orders assignment to community-based sheltered educational housings, which are residential institution offering further social and work rehabilitation, combined with a court-ordered outpatient psychiatric treatment of low intensity. In case of low yet substantial risk, the court orders the pursuit of psychiatric inpatient treatment and the MDO is transferred to a low-security ward outside the prison, to continue intense psychiatric treatment but with minimal disciplinary coercion. However, if the violence risk remains high despite the intensive treatment of the forensic psychiatric clinic, the court retains the absence of the therapeutic benefit, and orders imprisonment in a regular penitentiary to guarantee public safety, combined with maintenance of psychiatric treatment of low intensity.

In this study, risk assessment was made using a widely used structured professional judgment approach, the *Historical Clinical Risk Management* HCR-20 v2 (16, 17). This observer-rated instrument includes 10 historical risk factors (past events, experiences, or conditions known to increase the risk for violence), 5 clinical factors (lack of insight, negative attitudes, symptoms, impulsivity, and unresponsiveness to treatment) and 5 risk management factors (feasibility of plans, exposure to destabilizers, personal support, refusal to follow treatment, stress). All 20 items are coded with a rating of 0 to 2, with a total score ranking from 0 to 40, higher scores indicating higher risk levels. To detect change in risk factors, Webster et al. (16) recommend repeated assessment every six to twelve months. The HCR-20 has been subject to extensive empirical testing and the HCR-20 shows good predictive validity of abstention from violence and sensitivity to change (17).

### Measures of treatment progress

Indicators of treatment progress included a range of variables collected routinely in the forensic psychiatric clinic to assess inmates’ evolution. These include a standardized assessment of protective factors, as well as indicators of social and work rehabilitation (participation in rehabilitation programs, social support network, escorted leaves) and therapeutic security (disciplinary measures, drug abstinence tests).

Protective factors were assessed with the complementary *Structured Assessment of Protective Factors for violence risk* (SAPROF (18, 19). It contains 17 protective factors organized into three scales: internal factors (such as intelligence, empathy, and self-control), motivational factors (including work, motivation for treatment, attitudes towards authority) and external factors (social network, professional care, living circumstances). Among these factors, fifteen are dynamic, making them valuable targets for assessing treatment outcomes. Items are coded on a three-point scale (0-2), reaching a total score from 0 to 34, higher scores indicating a higher level of protection.

A binary evaluation of the participation to educational and professional rehabilitation training, was performed by the teacher/trainer (1=yes, 0=no) at each time point for three variables. Refresher courses provide a review and update of the pre-custody level of formal education (i.e. mathematics, French and English languages, computer science). Maintenance work includes cleaning, meal service, and snack-coffee-tobacco delivery. In a second step, after having progressed in work autonomy, inmates may also work outside their unit in vocational training workshops (laundry, cleaning, bakery, cooking, and gardening).

Escorted leaves prior to discharge are used to test the MDO’s capacity of adjustment to the social life, they include visits of their future living place, shopping in the city, or family gatherings. The number of visits by professionals such as lawyers or charity members, as well as by family members or friends are relevant indicators of the offenders’ social support network outside custody.

Disciplinary measures such as solitary confinement, fine, warning, or media deprivation, are applied to punish antisocial behaviors, including refusal to co-operate, threatening or immoral attitude, violence, damage of materials, and substance use. This latter was detected via unexpected urine drug tests.

### Statistical analysis

Descriptive statistics were used for sample description. Age, length of COT prior to admission in Curabilis and HCR and SAPROF scores were treated as continuous variables. Gender, type of offense, disciplinary measures, participation (yes/no) in refresher courses, were treated as binary variable. Nationality, education (school drop-out/obligatory schooling/apprenticeship/high school, university), family status (single/separated-divorced-widowed/married/parenthood), psychiatric ICD-10 diagnosis and type of criminal offense were treated as ordinal variables.

Longitudinal variation over the five time points was assessed with mixed-effects linear regression for continuous variables, mixed-effects logistic regression for binary variables, and multinomial logistic regressions for nominal variables taking into account the repeated measures design. We preferred mixed-effect regression models to classical repeated measures ANOVA, because it takes into account an imbalanced and missing data set, including subject clustering. In a real-life observational study design, most MDO were included in several diagnostic groups as a function of their comorbidities. We analyzed the three most frequent diagnostic groups both individually (F20, F60B, F10) and as comorbidities (F20+F10 respectively F60B+F10). In addition, some inmates were inevitably discharged before the end of the five time points. Each MDO has a different number of repeated assessments. For missing data, ANOVA can only use listwise deletion, which could affect the design and reduce power substantially. In addition, mixed-effect models allow for simultaneous consideration of changes in the main variables (i.e. HCR-20 score over time) as well as differences in mean levels of this variable, as has been previously stated (20).

Multinomial logistic regression models were used to predict discharge locations at release using the following longitudinal predictors: HCR and SAPROF score changes, psychosocial rehabilitation (refresher courses, work attendance, prison visits and leaves) and security (disciplinary measures, drug abstinence). Linear regression models were used to predict HCR-20 score changes (dependent variable) with psychiatric diagnosis and nature of offence as independent variables.

Prior to analyses, the extracted data was checked for incorrect dates, discharge dates were completed for those MDO who left the clinic between the end of the two-year’s time frame and data extraction. MDO’s age and length of past COT history were calculated. Data cleaning also included binary recoding of the participants nationality (swiss/foreign), as well as merging of high-school degrees and university diplomas into one single educational level. The significance level was set at p<0.05. All statistical analyses were performed using Stata 18.0.

## Results

### Sample characteristics

Inmates are more often male (94%, 110/117), single (79.5%, 93/117), with foreign nationality (54.7%, 64/117). Their mean age is of 35.8 (10.6) years. Their level of formal education is rather low, 35.9% (42/117) have accomplished obligatory schooling, 23.1% (27/117) an apprenticeship, and merely 6.8% (8/117) have reached high school or university level. About one third of the participants (34.2%, 40/117) have dropped out from school because of conduct disorders, drug use or violent behavior.

Psychotic disorders (schizophrenia, delusional disorder) were present in 67.5% (n=79) of the offenders, 58.1% (n=68) suffered from comorbid substance use disorders (SUD) and 38.5% (n=45) had Cluster B personality disorders such as borderline, narcissistic, or antisocial personalities. Intellectual disability was present in 14.5% (n=17) of cases. Less than 10% had a diagnosis of paranoid or schizoid personality disorder (5.9%, n=7), mood disorders (5.1%, n=6) or paraphilias (5.9%, n=7). The majority 73.5% (n=82) of the 117 MDO presented with two (n=55) or three (n=27) diagnosis, while the presence of four diagnosis remained rare (n=4). In case of double diagnosis, the most frequent combination was psychotic disorder with comorbid SUD (F20+F10: 38.5%, n=45) and Cluster B personality disorder with comorbid SUD (F60B+F10: 26.5%, n=31). No pattern emerged for the triple diagnosis, those MDO presented with multiple combinations of disorders.

Regarding criminal offenses, the majority of the inmates have been convicted for physical violence (82%, n=97) (bodily harm such as aggression, assault, fight, murder) and for violation of property 70.9% (n=83) (such as robbery, organized fraud, or breach of trust). Drug-related offenses were retained in 60.7% (n=71) of cases, violation of domestic privacy (threats, sequestration, and kidnapping) in half of cases (51.3%, n=60), and violation against the forces of order in 39.3 (n=46) of cases. One third (29.1%, n=34) of cases were sex offenders. Violation of honor and privacy (26.5%, n=31), and violation of road traffic (23.1%, n=27) were reported in a quarter of cases. Deliberately setting fire (13.7%, n=16), illegal immigration (15.4%, n=18) and violation of gun law (15.4%, n=18) are the less frequent offenses in our sample.

**Table 1** illustrates the demographic characteristics, criminal offenses, scale scores and length of court-ordered treatment prior to admission, according to the three main diagnostic groups. As expected, MDO with Cluster B Personality disorders are admitted with higher level of psychopathy, longer past treatment history, higher risk and lower protection scores, compared to the other diagnostic groups. Statistical significance in group comparisons was not assessed since it would be biased, because most MDO presented with various combinations of two or more diagnoses.

**Table 1:** Sample characteristics at admission according to the main psychiatric diagnosis.

	Psychotic disorders (F20-F29)	Cluster B Personality disorders (F60.2-F60.4)	Substance use disorders (F10-F19)
	n=79	n=45	n=68
Age (years mean, SD)	35.8 (9.6)	33.3 (10.6)	34.4 (9.9)
Male gender (%)	76 (96.2%)	40 (88.9%)	64 (94.1%)
Foreign nationality (%)	46 (58.2%)	23 (51.1%)	37 (54.4%)
Marital status (single %)	64 (81.0%)	36 (80.0%)	55 (80.9%)
Education			
School drop-out	25 (31.6%)	18 (40.0%)	27 (39.7%)
Obligatory schooling	28 (35.4%)	14 (31.1%)	19 (27.9%)
Apprenticship	20 (25.3%)	9 (20.0%)	17 (25.0%)
High school, university	6 (7.6%)	4 (8.9%)	5 (7.4%)
Type of criminal offense (Swiss Penal Code %)			
Physical violence (art. 111-136)	68 (86.1%)	35 (77.8%)	55 (80.9%)
Property violation (art. 137-172)	57 (72.2%)	33 (73.3%)	53 (77.9%)
Drug trafficking (Lstup, art. 118-123)	48 (60.8%)	31 (68.9%)	49 (72.1%)
Threat, sequestration, kidnapping (art. 180-186)	40 (50.6%)	23 (51.1%)	38 (55.9%)
Violation forces of order (art. 285-295)	31 (39.2%)	20 (44.4%)	31 (45.6%)
Honor and privacy (art. 173-179)	22 (27.8%)	12 (26.7%)	18 (26.5%)
Sexual offense (art. 187-200)	17 (21.5%)	15 (33.3%)	20 (29.4%)
Road traffic laws (LCR, art. 90-99)	17 (21.5%)	13 (28.9%)	17 (25.0%)
Illegal immigration (Letr)	14 (17.7%)	6 (13.3%)	11 (16.2%)
Gun law violation (Larm)	13 (16.5%)	4 (8.9%)	12 (17.6%)
Arson (art.221-230)	13 (16.5%)	3 (6.7%)	9 (13.2%)
At admission			
COT prior to admission (months; mean, SD)	30.2 (39.9)	47.6 (47.6)	31.2 (36.2)
Psychopathy (PCL-R score 0-40; mean, SD)	13.3 (6.1)	19.7 (6.8)	16.2 (6.7)
Violence risk (HCR-20 score 0-40; mean, SD)	21.6 (6.5)	25.3 (5.5)	22.7 (6.5)
Protective factors (SAPROF 0-34; mean, SD)	14.9 (5.7)	13.7 (5.7)	15.5 (5.9)
Main discharge location	n=64	n=37	n=51
Low-security psychiatry wards (%)	18 (28.1%)	7 (18.9%)	16 (31.4%)
Sheltered educational housing (%)	22 (34.4%)	10 (27.0%)	20 (39.2%)
Return to prison (%)	15 (23.4%)	16 (43.2%)	9 (17.6%)
Country of origin (%)	6 (9.4%)	3 (8.1%)	4 (7.8%)
Prison release (%)	2 (3.1%)	-	1 (1.9%)
Death (%)	1 (1.6%)	1 (2.7%)	1 (1.9%)

### Longitudinal treatment progress

**Table 2** displays the significant changes in work rehabilitation, security management and protective factors over time. The latter, as assessed by the SAPROF scale, were of rather low intensity at admission, but increased progressively during the hospital stay.

**Table 2:** Treatment progress indicators.

Time points	Baseline	6 months	12 months	18 months	24 months
N	117	85	63	49	29
SAPROF (0-34)					
Mean (SD)^1^ Coeff^2^ [95% CI]	14.6 (5.6) 0.00	15.6 (5.7) 1.25* [0.24,2.25]	15.7 (5.2) 2.18** [1.06,3.31]	17.2 (6.4) 3.53** [2.29,4.77]	18.2 (5.4) 5.11** [3.58,6.63]
Maintenance (ward)					
Participation rate	83% (97)	80% (68)	66% (42)	65% (32)	62% (18)
OR [95% CI]	1.00	29.16** [2.97,286.05]	16.49* [1.71,159.05]	14.34* [1.39,147.90]	2.87 [0.13,62.99]
Refresher courses					
Participation rate	17% (20)	20% (17)	33% (21)	34% (17)	37% (11)
OR [95% CI]	1.00	2.1 [0.6,7.6]	6.8* [1.7,27.1]	6.2* [1.4,26.7]	12.6* [2.1, 74.2]
Visits professionals (nb)					
Mean (SD) IRR [95% CI]	1.7 (2.3) 0.00	2.1 (2.3) 1.60** [1.20,2.14]	1.4 (1.6) 1.20 [0.86,1.69]	1.7 (2.3) 1.31 [0.91,1.90]	2.1 (2.4) 1.83* [1.20,2.79]
Positive drug test					
Rate	6% (7)	9% (8)	14% (9)	8% (4)	10% (3)
IRR [95% CI]	0.00	2.22 [0.43,11.4]	10.25* [1.72,61.07]	2.82 [0.37,21.39]	9.43* [1.05,84.74]
HCR-20 (0-40)					
Mean (SD) Coeff [95% CI]	22.6 (6.5) 0.00	21.1 (7.1) - 1.56* [-2.69, -0.43]	20.7 (7.1) - 1.92** [-3.19, -0.65]	18.4 (7.2) - 4.24** [-5.64, -2.85]	16.5 (7.3) - 6.12** [-7.84, -4.41]

^1^Observed mean and standard deviation without consideration of the repeated measures design.

^2^Significant coefficients are marked in bold (*p<0.05, **p<0.01).

The majority (83%) of MDO participated in maintenance work in the ward at their arrival. This participation rate progressively decreased to reach 65%, then remained stable after 18 months. Inversely, inmates showed a low participation rate of 17% in the refresher courses at admission, but this percentage increased after 12 months to nearly double after 24 months (37%). More than half of the MDO participated in vocational training during their stay (56%, n=65). For the remaining other half, 34% (n=40) were not able to work during the entire duration of their stay, and 10% (n=12) worked irregularly.

Escorted leaves remained stable over time, 75% of the inmates benefited from 2 to 3 leaves after a mean period of 18 months to prepare discharge. Regarding the number of external visits, inmates received a mean of 2 visits from professionals, namely their lawyers, and these visits took place at admission and the end of their stay. Family and friend visits were more frequent with a mean of 3.4 visits (SD=6.1) without significant change over time. Half of the inmates (51%) received between 3 and 30 visits during their stay, and the other half (49%) received no family-friends visits at all.

Disciplinary measures were rare (mean=0.7, SD=1.4) and did not evolve over time. 75% of the MDO received one, but only 7% received up to eight penalties during their stay. The rate of positive drug tests was very low (6%) at admission. Significant increases in its occurrence were observed at 12 (14%), and 24 months of stay (10%).

### Discharge locations at release

By the end of the study, 94 of the 117 inmates were released. Among them, 59% were either transferred into sheltered educational housing (n=30), or open low-security psychiatry wards (n=25). 28% of the inmates returned to regular prisons (n=26). 10% of the inmates were transferred to their country of origin (n=9) for treatment follow-up in outpatient settings or psychiatric hospitals, 2% were released conditionally without further treatment (n=2). Two participants died during the period of observation (1 suicide).

Interestingly, the SAPROF scores did not significantly predict the three main locations at discharge. Likewise, none of the significant treatment progress indicators (refresher courses, maintenance work, vocational training, escorted leaves, number of visits, disciplinary measures, or drug tests) predicted locations at discharge.

**Figure 1:**
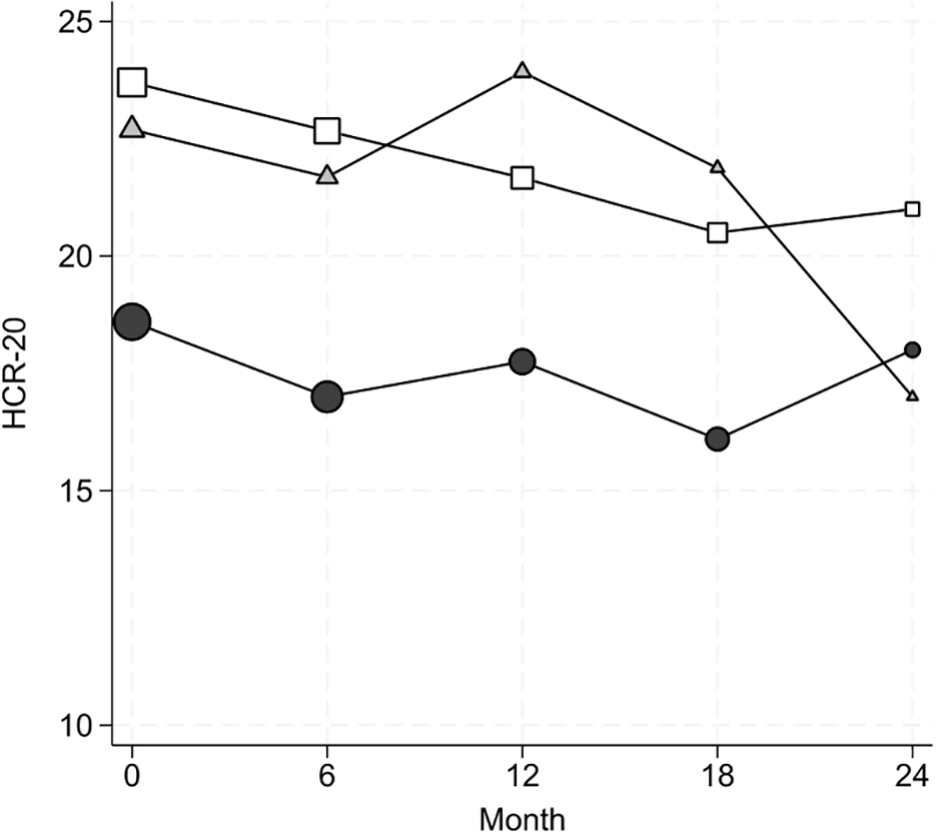
Evolution of HCR-20 scores as a function of location at discharge. The symbol size is proportional to the number of observations. Dots = low security psychiatry ward, Squares=sheltered educational housing, Triangles=prison.

The three discharge locations were predicted solely by the level of violence risk. MDO with lower HCR-20 scores at admission (RRR=0.88 [0.80, 0.97], p=0.011) have the best chances to be admitted to low-security psychiatry wards (**Figure 1**). When diagnostic groups are accounted for, two opposite patterns were detected. Higher HCR-20 scores at admission increased the chances to be released in sheltered education housing in inmates with psychotic disorders (F20-F29), rather than return to prison (RRR=4.36 [1.20, 15.81], p=0.025). In MDO with Cluster B personality disorders, higher HCR-20 scores at admission were associated with increased the probability to return to prison, rather than to sheltered education housing (RRR=0.11 [0.02, 0.51], p=0.005).

### Risk evolution according to diagnosis and offense

Overall, the risk of violence, as assessed by the HCR-20 scale, continuously decreased over two years (**Table 2**). The speed of decrease doubled after 18 months of stay.

**Figure 2:**
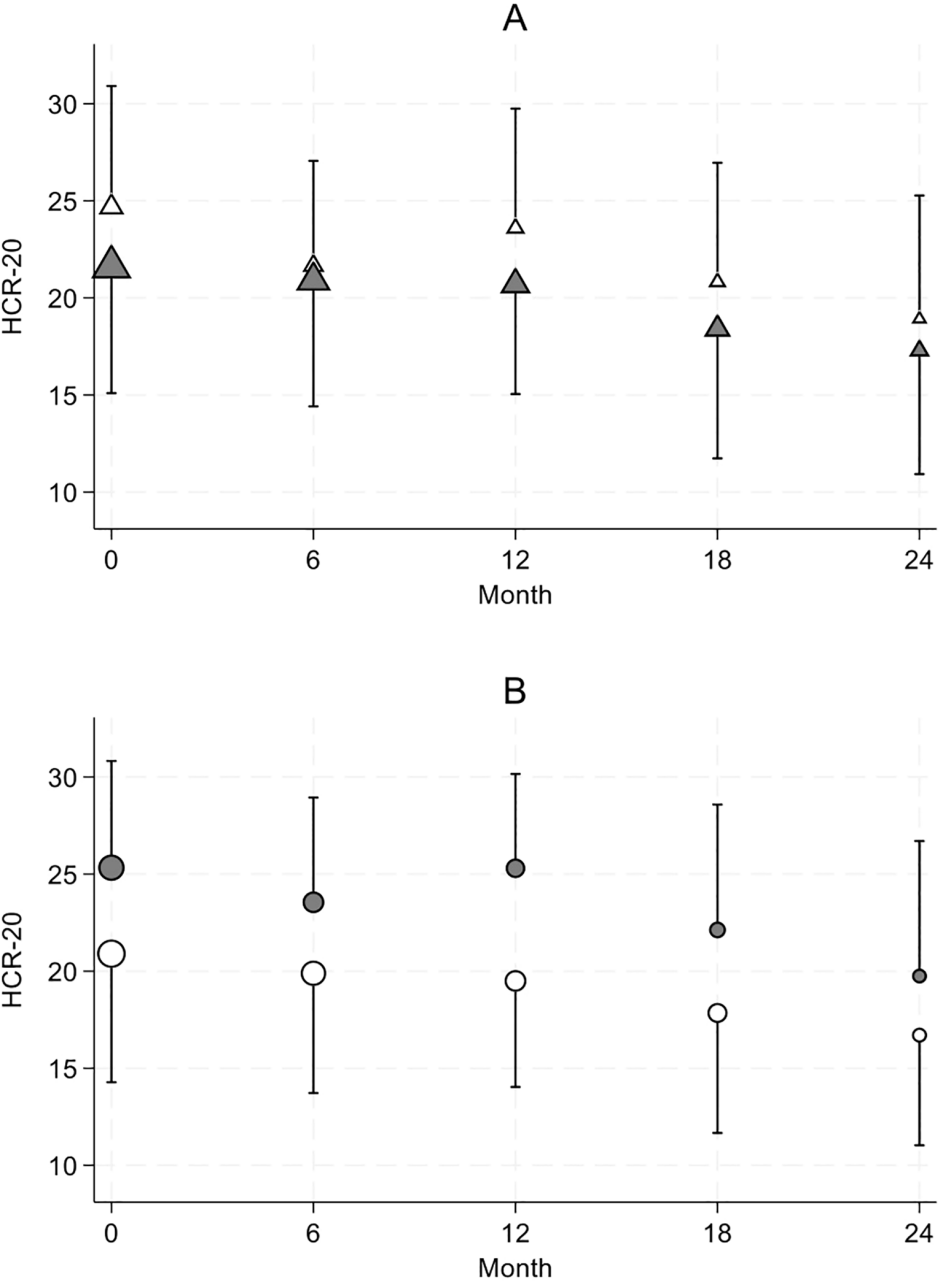
ICD-10 diagnosis related patterns of HCR-20 score evolution. The symbol size is proportional to the number of observations. Results are presented as mean ± one SD. **(A)** HCR-20 scores in Cluster B personality disorder (white triangle) versus all others diagnosis (grey triangle). **(B)** HCR-20 scores in psychotic disorder (white circle) versus all others diagnoses (grey circle).

The psychiatric diagnosis had a clear impact on the level of risk reduction. As shown in **Figure 2**, in mixed-effects regression models with diagnosis and time as covariates, MDO with antisocial or borderline personality disorders (F60.2-F60.4) displayed consistently HCR-20 higher risk scores (mean=25.3, SD=5.5) over time in comparison to the other MDO (coeff=4.43 [2.19,6.66], p<0.001, no interaction effect). Importantly, SUD comorbidity of personality disorders has no significant impact on the HCR-20 score evolution over time. Regarding inmates with psychotic disorders (F20-F29), their HCR-20 scores were consistently lower compared to the other MDO without time x diagnosis interaction (mean=21.5, SD=6.5; coeff=-3.07 [-5.49, -0.65], p=0.013). This pattern persisted independently of SUD comorbidity.

Offense types equally impact risk reduction. **Table 3** shows significant effects of the four most frequent types of offense (>50% of the sample). MDO treated for a crime without interpersonal violence, such as property violation, showed a more favorable outcome with a faster and stronger reduction of HCR-20 scores after 12 months (coeff=-4.30 [-7.12, -1.48], p=0.003) that persisted after 18 months of COT (coeff=-3.78 [-6.80, -0.76], p=0.014) compared to the other offenses as documented by a significant offense x time interaction effect. More concretely, as shown by the negative coefficient, their mean HCR-20 scores drop from 22.6 at baseline to 18.3 after 12 months of treatment and remained at 18.8 after 18 months. No significant main or interaction effect was observed for physical violence. In threat sentenced MDOs, compared to the HCR scores of the other MDOs, the risk level remained consistently higher over time (coeff=3.16 [0.91, 5.42], p=0.006), without significant time, diagnosis or offense interactions.

**Table 3:** Offense-related patterns of HCR-20 score reduction.

N=117 inmates measured 343 times	HCR-20 violence risk		
Baseline score: mean (SD)	22.6 (6.5)		
Property violation (art. 137-172)	Coefficient^1^	p	[95% CI]
Time X property violation			
6 months	- 2.31	0.066	[-4.79, 0.16]
12 months	- 4.30**	**0.003**	[-7.12, -1.48]
18 months	- 3.78*	**0.014**	[-6.80, -0.76]
24 months	- 2.80	0.177	[-6.86, 1.26]
Threat, sequestration, kidnapping (art. 180-186)			
Threat, sequestration, kidnapping	3.17**	**0.006**	[0.91, 5.42]
Drug trafficking (art. 118-123)			
Personality disorder (F60-B)	7.04**	**< 0.001**	[-3.53, 10.6]
Drug trafficking	6.46**	**< 0.001**	[3.87, 9.06]
Personality dis. X drug trafficking	- 5.05*	**0.024**	[-9.42, -0.67]
Time			
6 months	- 0.40	0.707	[-2.46,1.67]
12 months	- 0.69	0.553	[-2.96, 1.58]
18 months	- 3.30**	**0.008**	[-5.73, -0.87]
24 months	- 4.73**	**0.005**	[-8.01, -1.45]
Substance use disorder (F10-F19)	1.64	0.346	[-1.77, 5.06]
Drug trafficking	8.31**	**<0.001**	[5.04, 11.59]
SUD x drug trafficking	- 5.18*	**0.024**	[-9.68, -0.68]
Time			
6 months	- 1.61	0.162	[-3.87, 0.65]
12 months	- 1.18	0.367	[-3.74, 1.38]
18 months	- 3.56*	**0.015**	[-6.43,-0.68]
24 months	- 5.28**	**0.003**	[-8.71, -1.84]

^1^Significant coefficients are marked in bold (*p<0.05, **p<0.01). The coefficient unit corresponds to the actual HCR-20 scores changes, for example in MDO with property violation, their mean score drops from 22.6 to 18.3 after 12 months (coeff = - 4.30) and remains at 18.8 after 18 months.

In contrast, drug offenses show significant offense x diagnosis as well as offense x time interactions. HCR-20 scores are increased by drug trafficking from 16.2 (5.3) to 21.4 (5.3) in the absence of personality disorders. Yet, in the presence of personality disorders, HCR scores remain equally high with or without drug offenses, 23.8 (5.2) respectively 23.1 (5.3). The risk related to Cluster B personality disorder prevails over the drug offense-related risk. Regarding time interaction, the regression model adjusted for both drug trafficking and personality disorders, shows a slower HCR-20 decline only after 18 months of treatment. HCR-20 reduction also requires as much as 18 months of COT in a model adjusted for drug trafficking and SUD. HCR-20 scores are significantly higher in drug trafficking from 17.2 (5.5) to 24.2 (5.5) in the absence of SUD. This difference remains significant in the presence of SUD, with 19.8 (5.5) respectively 21.6 (5.4). As mentioned, SUD has no significant impact on HCR-20 scores.

## Discussion

The two-year longitudinal study assessed the determinants of the evolution of 117 MDO convicted to COT in a medium-security forensic psychiatric hospital. MDOs mainly presented with psychotic or Cluster B personality disorders, as well as comorbid SUD and for the majority, their convictions included physical violence, violation of property and domestic privacy, as well as drug-related offenses.

Our findings provide new insights on the relationship between psychiatric diagnosis, criminological factors and treatment outcome for MDO. The discharge location was predicted only by the HCR-20 patterns at admission. The other detention-related variables studied here, namely SAPROF levels at admission, participation in the work rehabilitation programs, security coercion, or the MDO’s external social network, did not impact on the final choice at location at discharge. Lower levels of violence risk are associated with discharge into low-security psychiatry wards independently of the psychiatric diagnosis. Higher initial HCR-20 scores increase the chances to be admitted in sheltered educational housing rather than return to traditional custody for inmates with psychotic disorders, but the contrary is true for inmates with Cluster B personality disorders. Second, a closer look on risk mitigation confirms both diagnosis-specific and offense-related patterns, as well as a significant offense-diagnosis interaction on violence risk decrease during inpatient COT. HCR-20 scores remained higher over time in Cluster B personality disorders and in MDO sentenced for property violation, threat, or drug offenses. In MDO sentenced for drug trafficking, favorable HCR-20 reduction is slowed down in Cluster B personality or SUD.

### Forensic psychiatry as a valid alternative to prison

Our results confirm the European Psychiatric Association guidance (2) which recommends forensic-psychiatric care for MDO rather than prison incarceration alone. Out of the 94 MDO who ended their stay, 55 were released to less restrictive settings after forensic treatment, compared to 26 who returned to the regular prisons. During their stay, inmates benefited from intensive inpatient court-ordered psychiatric treatment, in addition to traditional custody work rehabilitation. Indeed, more than 80% of the MDO invested maintenance work in the prison ward. This participation rate gradually dropped to 65% after 18 months of stay. Subsequently, after about 12 to 18 months, MDO significantly increased their participation in refresher courses to update their pre-custody level of formal education.

The secure prison-based environment guarantees the drug-free and violence-free environment essential for a treatment aiming the acquisition of prosocial behaviors. Prison populations are particularly affected by substance use in Europe (21). In-prison drug use is associated with addictive behaviors before imprisonment (22). In our sample, SUD was present in 58% of cases at admission and 60% had been convicted for drug trafficking. Nevertheless, substance use during their stay was rare and recorded in merely 6 to 14% of the inmates with a peak at 12 and 24 months of stay. Interestingly, these two peaks correspond to the timing of the annual renewal of their court-order that could induce significant levels of distress. The low level of drug use during their stay in Curabilis shows how inclusion of addiction management is a vital treatment component for MDO. Likewise, the use of disciplinary measures remained very limited. 75% of the inmates received only one penalty during their stay. Therapeutic community treatments are known to favor a prosocial climate through mutual peer monitoring among inmates in addition to the use of corrections (23).

### Risk assessment as forensic outcome

Overall, our sample displayed moderate violence risk as documented by the HCR-20 scores at baseline. Importantly, there was a significant decrease of 4.2 points after 18 months and 6.1 points after 24 months of treatment. The observed decrease is similar or stronger than in other high-security hospitals, which reported a drop of 1.5 to 4.5 points of the total HCR-20 score during comparable treatment length (20, 24). O. Shea et al. (9) found that HCR-20 total scores reduced by an average of 0.42 points per assessment. In our study, the total score reduction was higher, with an average decrease of 1.53 points per assessment (range 0.36 to 2.32). Importantly, the clinical significance of violence risk decrease over the course of the treatment cannot be established since the HCR-20 assessment is based on a structured professional judgment model without a widely accepted cut-off score for the HCR-20 risk scale. Subsequently, HCR-20 changes become evident only in group comparisons between *a priori* defined clinical groups (9).

Our findings also provide additional insights on the link between the risk level during COT and location at discharge. One third of the MDO were transferred to sheltered education housing, another third was referred to open low-security psychiatry wards and only a third returned to regular prison. Among the variables studied, only the HCR-20 score at admission was a significant predictor of transfer to low-security psychiatric wards. Studies on this issue remain quite rare. We previously reported that discharge locations in COT depend on pretreatment rather than treatment-related characteristics (7). In particular, younger age and conviction for property violation rather than physical violence increased the chances to be discharged to sheltered educational housing. Longer pre-treatment duration of COT, personality disorder diagnosis, and conviction for sexual offense increased the risk of return to prison (7). In the present study, low violence risk at admission was the only determinant of discharge locations. This observation is consistent with the conclusions of Probst et al. (25) who stressed the need to consider both pretreatment and treatment-related variables to identify the patients at risk of criminal recidivism after discharge from forensic treatment.

Importantly, the association between HCR-20 and location at discharge was diagnosis-dependent in the present sample. The persistence of higher HCR-20 scores was associated with more frequent institutionalization in sheltered education housing for psychotic MDO but also with return to prison for those with Cluster B personality disorders. In psychotic disorders, after treatment initiation the overall prevalence of violence drops gradually to rates close to those of the general population (26). It is thus likely that the persistence of increased violence risk in this subgroup leads to the decision of a long term stay in sheltered educational housing instead of low security psychiatric wards. However, return to prison was not considered as a credible alternative for these patients who often suffered from highly debilitating symptoms. In contrast, MDO with personality disorders are known to be treatment-resistant with little prospect of recovery or release while remaining at high risk of reoffending (3). In a previous study, Jeandarme et al. (3) concluded that because they present more incidents and are less likely to be resocialized, these MDO need higher security settings with long-stay units offering coercive measures and informal care. The present study confirms these findings. Even though levels of psychopathy are low overall, the Cluster B personality disorder subgroup showed higher initial PCL-R scores, persistently higher HCR-20 violence risk scores, and increased likelihood to return to prison. Future studies will have to study additional variables to explain the treatment resistance in these MDO group. They might include detailed monitoring of pharmacological treatment, as well as intellectual and social cognition deficits.

An additional objective of the present study was to analyze the impact of diagnoses and type of offense on the longitudinal evolution of violence risk in COT. As one could expect, MDO with antisocial or borderline personality disorders have significantly and systematically higher risk from admission until discharge over the five timepoints, compared to other MDO. Previous evidence has shown that externalizing individuals, who manifest a developmental trajectory from severe childhood conduct disorder through early onset substance use to adult antisocial or borderline personality disorder, display increased risk of violence and criminal recidivism (27). While some authors have suggested that violent individuals with schizophrenia and antisocial personality disorder share common emotion processing deficits such as facial affect recognition (28), others have identified distinct neuronal mechanisms underlying affective theory of mind in violent antisocial personality disorders and schizophrenia (29). The present data imply that despite comparable inpatient COT, MDO patients with personality disorders are less responsive to care compared to psychotic patients at least in the context of a medium-security hospital.

Dramatic and erratic personality disorders are frequently associated with SUD. SUD comorbidity also heightened the likelihood of criminality in schizophrenia in some studies (30–32). Likewise, comorbid alcohol and drug substance use have been shown to enhance violence in antisocial/borderline personality disorder in secure hospital inpatients (27). The present findings do not confirm these reports. In our sample and independently of the diagnosis, comorbid SUD did not impact on the evolution of HCR-20 scores over time. One possible explanation for this discrepancy may reside to the legal context of Switzerland. The Swiss Criminal Code considers two distinct COTs for MDO with primary single SUD versus MDO with primary mental disorders and SUD comorbidity. It is thus plausible that the MDO in the present sample showed mild SUD that is also in line with the very low rates of drug consumption in Curabilis. Our data are consistent with the work by Short and collaborators (33) who postulated that the increased risk of violent offending in schizophrenia cannot be solely attributed to the effects of comorbid substance use.

In agreement with the idea that offenses involving interpersonal risk issues score higher in risk assessment (16), MDO convicted for property violation showed the most favorable risk reduction over time after merely 12 months of COT. In contrast, MDO convicted for threat, sequestration, or kidnapping, remain consistently at higher risk over time independently of the duration of their stay. Drug offense sentenced MDO revealed the most treatment resistant sub-group, independently of their Cluster B personality or SUD disorder. As has been previously described, criminal desistance in those drug offenders needs both disease recovery and the development of a prosocial identity (34). Desistance occurs when the intrinsic motivation to change (inner change agent) is present. The prospect for successful desistance relies on the individual’s – most probably pre-treatment – personal and social resources (35, 36). Further research is clearly warranted to address the underlying psychological processes at work initiating long-term desistance in MDO sentenced for drug-related crimes.

### Strengths and limitations

Strengths of the present study include the assessment of a large homogeneous sample of MDO in the only Swiss forensic psychiatry clinic for the French and Italian speaking parts of Switzerland. All participants were submitted to the same COT based on therapeutic community approaches. Moreover, our assessment included both detention-related and clinical variables that reflect the multidisciplinary aspect of the proposed care programs.

Several limitations should be considered when interpreting our findings. First, and to be close to real life, the diagnosis of personality disorders was made without standardized questionnaires by psychiatric experts. SUD diagnosis concerned only MDO with comorbid disorders of mild severity, since offenders with a primary diagnosis of SUD are convicted to a distinct addiction-focused COT according to the Swiss Criminal Code. Likewise, since scale ratings from routine clinical practice were used, it is likely that assessments were completed by different raters over time. We were not thus able to ascertain inter-reliability ratings for different raters. We cannot exclude that raters may consciously or unconsciously rate patients at lower risk over time to reflect assumed target progress. Second, this study has no medico-economic arm so that we cannot comment on the cost/effectiveness of the care programs in Curabilis, in particular for inmates with personality disorders. Third, we assessed participant rates in education and professional rehabilitation, however no routine assessment of the MDO’s individual treatment motivation and commitment was available to allow for adjustment of this variable. Fourth, due to the limited number of cases, we restricted our analysis to the HCR-20 total score. Consequently, no distinction was made between the historical factors that are static and irreversible, and dynamic clinical factors that are treatable. The use of the total score instead of the summary risk ratings (low, moderate, or high risk) could reduce the effect size of our results. The HCR-20 version 3 would have been an ideal alternative. It has been shown to be particularly relevant to conditional release decision-making because its contextual scenario planning includes the MDO’s individual motivations for violence (37). Unfortunately, the French Version 3 has not yet been implemented in Curabilis in routine clinical practice.

## Conclusion

The present study explores inpatient forensic treatment progress over time in a representative sample of imprisoned Swiss MDO. Post-treatment discharge locations are predicted solely by the pre-treatment violence risk level. Independently of their diagnosis and type of offense, MDO with lower risk levels at baseline have the best chances to be released from prison into low security psychiatry wards. In MDO with higher risk levels, only inmates with psychotic disorders are released from prison into sheltered educational housing. Violence risk reduction is mostly observed in MDO convicted for crimes without interpersonal violence, but not in those convicted for threat and sequestration crimes, or drug trafficking. The presence of Cluster B personality disorders is associated with the worst prognosis in our care setting.

Our observations point to the need of improving definitions of admission/refusal criteria for COT treatment and confirm the need of systematic risk assessment trajectories to prevent recidivism, taking into account the MDO diagnosis-specific needs. Indeed, MDO with psychotic disorders who do benefit from pharmaceutical medication in the context of their forensic therapeutic community treatment are clearly indicated for COT. In contract, MDO with Cluster B personality disorders may rather benefit from higher security settings with long-stay units offering coercive measures and informal care rather than therapeutic community treatment. Future studies in larger samples addressing both cost/effectiveness and the issue of static/dynamic risk distinction are needed to identify the predictors of clinical trajectories and define MDO subgroups that can optimally benefit from COT.

## Data Availability

The raw data supporting the conclusions of this article will be made available by the authors, without undue reservation.
